# 血液病患者异基因造血干细胞移植后弓形虫感染2例报告及文献复习

**DOI:** 10.3760/cma.j.issn.0253-2727.2023.10.013

**Published:** 2023-10

**Authors:** 卫华 翟, 利宁 张, 佳丽 王, 祎 何, 尔烈 姜, 四洲 冯, 明哲 韩

**Affiliations:** 1 中国医学科学院血液病医院（中国医学科学院血液学研究所），实验血液学国家重点实验室，国家血液系统疾病临床医学研究中心，细胞生态海河实验室，天津 300020 State Key Laboratory of Experimental Hematology, National Clinical Research Centre for Blood Diseases, Haihe Laboratory of Cell Ecosystem, Tianjin Institutes of Health Science, Institute of Hematology and Blood Diseases Hospital, Chinese Academy of Medical Science & Peking Union Medical College, Tianjin 300020, China; 2 天津医学健康研究院，天津 301600 Tianjin Institutes of Health Science, Tianjin 301600, China

弓形虫病是一种少见但可能致命的机会性寄生虫感染，可发生于异基因造血干细胞移植（allo-HSCT）受者。移植后任何时间都可能发病，中位发病时间为术后2个月。弓形虫肺部感染非常罕见，预后非常差，病死率极高，弓形虫病的影像学表现与耶氏肺孢子菌肺炎（PJP）类似；确诊通常需要证实组织或体液中存在速殖子。弓形虫感染无特征性的临床症状及体征、病原体镜检阳性率不高、形态难以鉴别，因此需要提高警惕，更强调预防。对于allo-HSCT患者，推荐从植活到整个免疫抑制治疗期间持续给予复方磺胺甲唑（SMZco）进行预防性治疗。本文报道两例通过宏基因组二代测序（mNGS）技术确诊的移植后弓形虫感染病例并进行文献复习。

## 病例资料

例1，男，48岁，2018年10月诊断为“急性髓系白血病（AML）-M_2a_（伴FLT3-ITD阳性）”。行DA方案（柔红霉素+阿糖胞苷）诱导化疗，并入组奎扎替尼临床试验，2个疗程后达完全缓解（CR）。后予巩固化疗4个疗程，持续CR。患者与其女儿HLA配型高分辨7/10位点相合，行单倍体外周血造血干细胞移植（haplo-PBSCT）。回输前给予1周的缬更昔洛韦450 mg/d、SMZco 976 mg每日2次进行巨细胞病毒（CMV）及耶氏肺孢子菌感染预防。预处理方案为清髓性改良BuCy方案，移植物抗宿主病（GVHD）预防采用他克莫司（FK506）+霉酚酸酯（MMF）+短疗程甲氨蝶呤（MTX）+抗胸腺细胞球蛋白（ATG）。累计回输单个核细胞（MNC）11.17×10^8^/kg、CD34^+^细胞1.42×10^6^/kg。+10 d粒系植活；骨髓CR，流式细胞术检测AML残留病（MRD-AML）阴性，FLT3-ITD等位基因比率0，染色体荧光原位杂交（FISH）示XX信号99.8％。+16 d开始出现间断低热，先后予美罗培南、莫西沙星、头孢哌酮舒巴坦抗细菌，伏立康唑预防真菌感染，监测C反应蛋白（CRP）轻度增高、CMV-DNA<1 000拷贝/ml、EB病毒DNA<1 000拷贝/ml、血培养阴性。+33 d胸部CT示：左肺尖、两肺胸膜下多发小结节影。+38 d开始发热较前频繁，无咳嗽、咳痰等症状，指脉血氧饱和度（SpO_2_）98％～100％，经验性加用利奈唑胺，试图覆盖可能的阳性菌或结核菌感染。+42 d仍有发热，结核杆菌斑点试验（T-SPOT）结果阴性，除外结核菌感染，无阳性菌感染证据，停用利奈唑胺。静脉血宏基因组二代测序（mNGS）结果示：人巨细胞病毒465总序列数（total reads）（基因组覆盖度14.8％、物种相对丰度100％），刚地弓形虫225 total reads（基因组覆盖度0.026％、物种相对丰度100％）。不除外巨细胞病毒及弓形虫感染，予加用膦甲酸钠3 g每12 h 1次静脉滴注、SMZco 976 mg每8 h 1次。追问患者及家属均无弓形虫感染史及猫狗接触史，未吃未煮熟的蔬菜及肉类。患者发热较前频繁，每日发热1～2次。+43 d复查胸部CT示：两肺可见多发小点状影、小树芽及索条，原左肺尖、两肺胸膜下多发小结节影仍可见。+46 d体温38.1 °C、SpO_2_降至90％，未感胸闷憋气。胸部CT（图1）示：两肺内小结节影增大、部分呈小斑片影，磨玻璃影增多，小叶间隔增厚；PJP可能性大。停用伏立康唑，改用卡泊芬净70 mg/d静脉滴注联合SMZco 976 mg每8 h 1次治疗，甲泼尼龙40 mg每12 h 1次静脉滴注。经支气管肺活检，快速现场评价（ROSE）可见纤维母细胞满视野、弓形虫包涵体（图2）。肺泡灌洗液及肺组织mNGS结果：刚地弓形虫分别为49 000、23 907 total reads（基因组覆盖度0.82％、物种相对丰度98.11％）。明确诊断：AML-M_2a_单倍体移植后刚地弓形虫肺炎。后患者诉胸闷、憋气，高通量吸氧下SpO_2_ 89％～92％，改为无创呼吸机CPAP模式辅助通气，SpO_2_升至96％～97％。+47 d病情加重，SpO_2_ 75％～80％。动脉血血气酸碱分析：血液酸碱度（pH）7.49，二氧化碳分压（PCO_2_）28.4 mmHg，碳酸氢盐（cHCO_3_）21.1 mmol/L。生化：ALT 224.7 U/L，AST 202.3 U/L、肌红蛋白MYO>500 µg/L、肌酸激酶同功酶CKMB 5.7 µg/L。患者憋气明显，咳嗽、咯痰，痰中带少量血丝。查体：双肺呼吸音粗、满布干湿啰音，心率150次/min。+48 d监测SpO_2_ 25％～60％、轻度活动后降至16％，病情危重，自动出院。

**图1:**
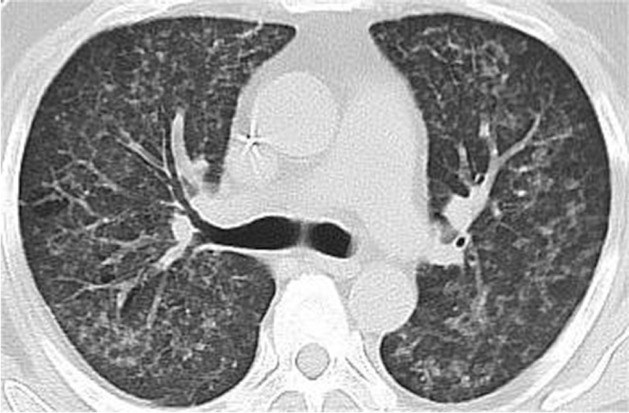
急性髓系白血病患者异基因造血干细胞移植后弓形虫感染胸部CT影像（两肺感染性病变，两肺内小结节影增大、部分呈小斑片影，磨玻璃影增多，小叶间隔增厚）

**图2:**
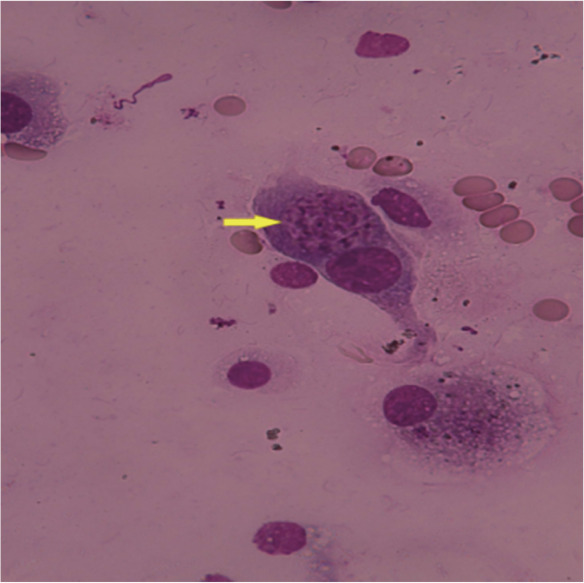
急性髓系白血病患者异基因造血干细胞移植后弓形虫感染快速现场评价结果（高倍，Diff-Quik染色，箭头所示弓形虫细胞内速殖子呈圆形、椭圆形或香蕉形，末端稍尖，可见1～2个核）

例2，女，40岁，2020年7月发现颈部、腋下、腹股沟淋巴结肿大，伴低热，2020年9月切检颈部右侧淋巴结，病理诊断间变大细胞T-细胞淋巴瘤（ALK阴性）。2020年11月予维布妥西单抗+环磷酰胺+吡柔比星+泼尼松化疗6个周期。2021年4月行自体外周血干细胞采集，2021年5月24日行自体移植。+2个月出现颈部淋巴结肿大，行颈部左侧淋巴结切检，经病理检查，明确诊断间变大细胞T-细胞淋巴瘤，ALK阴性（Ⅲ期B自体造血干细胞移植后复发）。先后予沙利度胺、米托蒽醌脂质体治疗2个周期，米托蒽醌脂质体+西达苯胺治疗1个周期。患者与其胞妹HLA配型6/12位点相合，行haplo-PBSCT。2022年7月28日PET-CT检查示：脑部+体部显像未见典型恶性肿瘤征象，符合淋巴瘤治疗后图像特征，考虑治疗有效，疾病缓解。回输前给予1周的缬更昔洛韦、SMZco进行CMV及耶氏肺孢子菌感染预防。2022年8月25起行预处理，方案为塞替派+Bu+Cy。GVHD预防为FK506+MMF+短疗程MTX+ATG。2022年9月2日回输供者MNC 8×10^8^/kg、CD34^+^细胞3.52×10^6^/kg。+10 d粒系植活；+11 d脱离血小板输注。+22 d出现反复发热，体温最高38.5 °C，伴畏寒不适，血培养阴性，mNGS检出刚地弓形虫1 150 total reads（基因组覆盖度0.092 4％、物种相对丰度100％），弓形虫-DNA阴性、IgG弱阳性、IgM阴性，完善头部MRI及消化系、泌尿系彩超未见包囊。请传染病医院会诊，诊断移植后弓形虫感染，给予口服SMZco 1 952 mg每8 h 1次，患者未再发热。+2个月复查mNGS未见弓形虫序列，弓形虫-DNA阴性、IgG阴性、IgM阴性，胸部CT未见感染病灶，予SMZco逐渐减量至976 mg/d维持治疗。

## 讨论及文献复习

两例患者均系高危恶性血液病allo-HSCT后发热，无皮疹、恶心呕吐、腹痛腹泻等GVHD表现，可以除外免疫因素导致的发热。由于细胞免疫和体液免疫受损，在造血重建后，感染仍是导致移植并发症和死亡的一个重要原因。许多细菌均可导致感染，包括军团菌属、诺卡菌属和放线菌属等；此外，还有CMV、EB病毒、结核菌、PJP、真菌及少见病原体等感染可能。例1经过调整抗感染治疗，仍反复发热，常规化验无明显阳性结果，进一步行血mNGS结果提示CMV、弓形虫感染，此后出现肺炎，肺CT提示PJP，最后经纤维支气管镜行肺活检，ROSE见弓形体包涵体及急性纤维组织机化表现，肺泡灌洗液及肺组织mNGS证实为弓形虫肺炎，虽然给予SMZco治疗，但是患者病情进展迅速、死于呼吸衰竭。例2是移植后反复发热，早期行血mNGS检出弓形虫，及时给予足量SMZco治疗，推测因为早发现、早诊断、早治疗，例2的弓形虫感染才得以控制，最终获得治愈。

弓形虫亦称弓浆虫，寄生于人类和多种动物的有核细胞，引起人畜共患的弓形虫病（toxoplasmosis）。特别在宿主免疫功能低下时致病，属机会致病原虫（opportunistic protozoa）。因滋养体似弓形或半月形而得名。其生活史分为中间宿主和终宿主，在中间宿主进行无性生殖，在终宿主进行有性生殖。终宿主为猫科动物，在猫肠绒上皮毛细胞内完成无性的裂体增殖和有性的配子生殖；中间宿主包括人类及多种动物，在中间宿主有核细胞内进行无性的内二芽殖增殖。弓形虫生活史中有5种主要形态：即滋养体（包括速殖子和缓殖子）、包囊、裂殖体、配子体和卵囊。但具有致病性和传播性的发育阶段为滋养体阶段，速殖子形成的假包囊阶段，缓殖子形成的包囊阶段，以及卵囊阶段。

弓形虫病是一种少见（<1％）但可能致命的机会性寄生虫感染，可发生于allo-HSCT患者[1]–[2]，尤其是流行地区。大多数弓形虫病都由再激活引起，罕见的供者来源感染也有报道。弓形虫感染最常表现为心肌炎或心肌病，但也可表现为脑脓肿、肺炎、脓胸或播散性感染[1]–[3]。移植后任何时间都可能发病，中位发病时间为术后2个月；高达10％的患者会在移植后1个月内出现感染再激活[4]。弓形虫肺炎表现为发热、呼吸困难和干咳，胸片通常显示网状结节浸润，弓形虫病的影像学表现与PJP类似[3]，确诊需要证实组织或液体中（如支气管肺泡灌洗液）存在速殖子[5]。除此之外可以进行弓形虫特异性抗体IgG/IgM检测、抗原检测、弓形虫DNA检测，但存在假阴性可能。外周血mNGS在血液病合并发热患者病原检出方面具有较高的临床认可率、敏感度及阴性预测值，有较高的临床应用价值[6]。虽然mNGS技术不能取代传统病原学诊断技术，但是，当传统检测手段不能明确致病微生物时，mNGS作为补充，可以指导精准治疗[7]。此外，经支气管镜介入诊断是获取肺部感染病原微生物证据以优化药物选择的重要方式之一。ROSE技术是一项实时伴随于取材过程的快速细胞学判读技术。例1是通过mNGS与ROSE技术结合确诊的，这一方法在疑、难、危、重，尤其传统病原微生物检测阴性的肺部感染诊断中将发挥越来越重要的作用[8]。

治疗弓形虫病的首选和备选方案大多包含磺胺嘧啶和乙胺嘧啶。如果无法迅速获得乙胺嘧啶，则应改用SMZco（无磺胺类过敏者）或阿托伐醌（有磺胺类过敏者）。通过两个病例对比及文献复习[1]–[3],[9]，弓形虫感染无特征性的临床症状及体征、病原体镜检阳性率不高、形态难以鉴别。因此，需要提高警惕，更强调对于移植后患者弓形虫感染的预防。由于担心发生骨髓抑制，通常在移植物植活后才开始SMZco预防性用药。对于allo-HSCT患者，推荐从植活到整个免疫抑制治疗期间持续给予SMZco进行预防性治疗[10]。

综上，这两例患者同为移植后弓形虫感染，但临床结局迥异，mNGS在少见病原微生物的检出中具有一定优势，我们建议对于反复发热患者，尽早送检mNGS，及时给予精准治疗，可以提高疗效。此外，我们强烈建议需警惕血液病患者allo-HSCT后弓形虫感染，移植物植活后尽早开始SMZco口服，是有效预防弓形虫感染的策略。
